# Progress in Medicine

**Published:** 1897-07-17

**Authors:** 


### Progress in Medicine.

### DISEASES OF THE DIGESTIVE ORGANS.

*(Continued from page 250.)*

**Peritoneum.**—Phantom tumours have often been regarded as hysterical phenomena pure and simple, but Arch. Donald[1] classifies them as follows: (*a*) Those produced in pelvic peritonitis by reflex irritation; (*b*) those occurring at the menopause; (*c*) those in young women with amenorrhœa and rapid increase of fat without known cause; (*d*) those which are purely hysterical. The most important, perhaps, are those which are associated with organic disease in the pelvis, or elsewhere; but in all of these we find the tympanitic note due to accumulation of gas in the intestine, and in most of them also localised contraction of the abdominal muscles, and an unusual deposit of fat in the abdominal wall and omentum.

Acute general peritonitis implies an inflammation coupled with a toxæmia; and, strictly speaking, there is no such thing as an idiopathic form of it. If the amount of toxins absorbed be very great, or the vital resistance of the body be low, inflammation may arise from causes too slight to be recognised. H. M. Stowe[2] divides the causes of infection into three groups, (*a*) those from the alimentary canal, (*b*) those from other contiguous organs, and (*c*) those from the blood. Numerous organisms, including the bacillus coli, which itself includes some fifty species, as he reckons them, may produce it. As long as the structure is normal the peritoneum can deal with considerable quantities of infective material, but when injured it easily succumbs to infection. The grave symptoms so often seen early in peritonitis are probably not due to the inflammation by itself, for this may turn out to be very limited. On the other hand free suppuration may end favourably, especially in children, with the formation of a sinus, and the remaining products may undergo more or less of organisation. The effusion varies like that of other serous cavities. In a recent number various cases of chylous ascites were reported; A. M. Stalkar[3] discusses one in which a sarcomatous growth of the mesentery cut off the lymphatics from the thoracic duct so that the fluid passed through the degenerating growth into the peritoneum. A similar process may occur in tuberculosis, while the more common type is that in which the thoracic duct is occluded and then ruptured. In cases like Dr. Stalker's the chyle is a perfect emulsion, but where the duct is ruptured numerous fat globules are present.

**The Liver.**—Sir Dyce Duckworth,[4] in speaking of cirrhosis, remarks that we have to do with a disease spread through every part of the body, and that while the force of it may fall in one person on the liver, in another it may affect the nervous system. Thus, in young women alcoholism produces more often peripheral neuritis than cirrhosis of the liver. He would classify cases of cirrhosis under three heads, viz.: (*a*) The atrophic form due to alcohol, with ascites and wasting, but showing little or no jaundice; (*b*) the hypertrophic form due to alcohol, with a less degree of ascites, but an equally fatal ending; (*c*) the hypertrophic form of bile duct origin, where is much jaundice but no ascites or history of alcohol. As to treatment, he insists on the absolute stoppage of all alcohol, though he sometimes allows it when the patient's recovery is hopeless to alleviate his sufferings. Early and repeated paracentesis is desirable till the later stages are reached, when tapping, unless urgently needed, is to be feared, as it is sometimes followed by cerebral symptoms and a typhoid state.

Chittenden, of Yale,[5] discusses the formation of gall stones, and argues that the concentration of the bile and the increase of cholesterin, though important factors, do not comprise all that is necessary for crystallisation and the formation of stone. We do not know the ultimate cause of the process, or that of the origin of the nucleus. He disbelieves in the possibility of reacting upon the salts so as to dissolve the stone, but a more likely method may be found by increasing the alkalinity of the bile and the peristaltic action when there is a free passage for that fluid through the ducts. Gilman Thompson also takes a pessimist view of the results of medical treatment, and merely recommends the relief of spasms by morphia, with draughts of spirits of chloroform and cherry laurel water. Olive oil had not proved to be of value in the trials he had made of it. For prophylaxis he forbids sugars and fats, cooked liver and brain, but recommends green vegetables and acid fruits with plenty of fluids, except aerated waters containing salts of lime, and alcohol in any form.

A curious variety of the disease is known as the ball-valve gall-stone. W. Osler,[6] in giving the history of three cases, notices the attacks of hepatic pain, the jaundice usually slight but increasing at times in intensity, and the paroxysm of intermittent fever known by the name of Charcot.

An important factor in the history of a gall-stone is the sphincter of the common duct, which, by the way, is not described in ordinary text-books, though Doyon has traced its afferent nerve-supply to the vagus, and its efferent to the splanchnic. The object of this sphincter is to fill the gall-bladder with bile and to permit an occasional outflow. Immediately behind this sphincter is a distended part of the tube, the ampulla of Vater. Thus a stone may be impacted and yet leave a channel for the passage of bile, or it may act as a ball-valve and prevent the outflow until the pressure behind it reaches a certain limit. It is probably this ball-valve action which produces the intermittent attacks which are so remarkable. The stasis of the bile affords an opportunity for the multiplication of microbes, to which the febrile symptoms are due. This infective cholangitis may go on for years without causing ulceration or suppuration in the ducts. Such cases of ball-valve gall-stones are often regarded as obstinate malaria, and, indeed the periodicity may be typical. They may be distinguished from abscess of the liver by the good condition of the patient between the attacks, the chronic course of the disease, and the absence of enlargement and tenderness. If suppuration should take place, then true pyæmic symptoms are found, the paroxysms are more frequent and irregular, and in the intervals the patient is no longer in good health. There is more tenderness and less jaundice, without fluctuations of intensity. As to treatment and prognosis, since some of the cases recover entirely, if the attacks are not increasing in frequency and severity, palliative measures are to be advised, for the operation is a much more serious one than for stones lying in the bladder. Where a downward progress is going on it may, however, be necessary to face this, and Kehr has recently recorded thirty operations with only two deaths.

Auscher and Lapicque[7] have investigated the formation of rubigin in pigmental cirrhosis, and find that it is formed from blood extravasated by an internal hæmorrhage. This undergoes a chemical change in the lymphatics, and is deposited in the various organs, though the latter take no active part in the formation of the pigment. There may possibly be other modes in which the pigment is formed, but this is the only form which has been proved to occur.

Simon Flexner[8] records a very rare complication of amœbic abscesses of the liver in the perforation of the inferior vena cava. This appears to have been noticed only once before. In one of the two patients whom he observed there were no intestinal lesions, though amœbæ were found in the abscess itself.

**Spleen.**—Two remarkable cases of enlarged spleen are worth noticing here. The first was in a woman under the care of Bonardi.[9] She had suckled a child for 22 months, and had suffered from influenza and metrorrhagia. The spleen became enlarged and hard, and, after seven years, hepatic cirrhosis followed with ascites. The examination of the blood showed—white C. 8,500, red C. 2,780,000, no microcytes. No history of malaria, syphilis, or alcohol was found out to throw light on this extraordinary complication. The other instance of splenic enlargement, recorded by M. L. Griffin,[10] appeared to follow an attack of typhoid 15 years before. The spleen reached down into the left iliac fossa, and for an inch and a half to the right of the umbilicus. The blood examination showed nothing abnormal in the white corpuscles, and the patient continued his daily labour, feeling in good health. The interest of the case is increased by his complete recovery, and the disappearance of the splenic tumour in 21 months under arsenic. Such a condition is, however, a source of great danger. Thus, in a recent case of an enlarged spleen, where the patient received a severe blow, rupture took place with severe hæmorrhage and collapse. Drs. Strange and Ware[11] opened the abdomen and removed the spleen, but the patient died in spite of repeated saline injections.

[1] Practitioner, March. [2] International Med. Mag., Feb. [3] Scottish Med. Chir. Jour., Mar. [4] Practitioner, March. [5] Med. Rec., Ap. 24. [6] Lancet, May 15. [7] Med. Week, Feb. 19. [8] Amer. J. Med. Sc., May. [9] B. Med. Jour., Feb. 27. [10] Dublin Jour. Med. Sc., April. [11] B. Med. Jour., May 1.

### DISEASES OF THE LUNGS.—TUBERCULOSIS.

(*Continued from page* 252.)

**Resection of Lung.**—Resection of lung or pneumectomy, continues Fowler, would appear at first glance to be a rational method of procedure in tuberculosis of the pulmonary structure. Surgeons who are accustomed to practise resections of the articulous extremities in tuberculous osteo-arthritis, and extirpation of the synovia in tuberculous synovitis—and who have witnessed the gratifying results following these operations—are at once impressed with a desire to institute radical operative methods in a disease which counts its victims by thousands yearly. The pulmonary structure, however, differs from all other structures in the body in its susceptibility to infection by means of the bacillus tuberculosis, and its anatomical peculiarities are such as to favour extension of infection and reinfection of parts whose vital resistance has been lowered by disturbances of nutrition from any cause.

Fowler proceeds to cite and summarise the recorded cases of Milton, Anthony, Tuffier (the first successful case of pneumectomy for pulmonary tuberculosis, 1892), and Lowson, and cites the experimental operations on lower animals of Gluck, Hans Schmidt, Block, Bivondi and Zaleharevitch. He states that Lowson operated as follows: An incision was made from the mid-sternum along the second rib through the pectoralis muscle nearly to the anterior axillary line. A downward cut was made, two inches long, from the inner end of this incision along the middle line of the sternum. The surfaces of the second and third ribs were thus exposed. These were excised after separation of the pleura. The pleura was then punctured by a trocar, which was connected by tubing to a Junker's inhaler bottle containing carbolic acid. Air was then slowly forced through the carbolic acid and into the thoracic cavity for the purpose of bringing about collapse of the lung. This process was not attended by dyspnœa or cyanosis. The pleural cavity was then opened freely, the lung detached from adhesions at its apex, transfixed in two places below the disease, and tied off with twisted silk thread. The wound was closed without drainage. The latter, however, proved to be an error. Hæmothorax was found to be present at the end of a month, which resulted in an empyema, and necessitated further interference. We cannot follow Fowler's interesting paper further, but he goes on to discuss the surgery of tuberculous lesions in the mediastinal space, and tuberculous pericarditis. Two cases, one of lobar pneumonia followed by gangrene of the lung, and one of liver and lung abscess, in both of which operation was successfully performed under the care of Surgeon-Captain D. M. Moir, I.M.S.,[15] are reported. Case 1: Lobar pneumonia; gangrene of the lung; operation; recovery. A man, aged 23 years, had an attack of acute pneumonia of the left lung at Jamalpur on February 13th, 1896. In the second week he suffered a good deal from diarrhœa and profuse perspiration. It was evident that septic absorption was going on, and the physical signs pointed to gangrene of the lung. The temperature was irregular, rising from normal to 103 deg. F. In the third week attacks of hæmoptysis supervened. The cough was very troublesome; the sputum was very offensive, copious, and coffee-coloured, with exhausting sweats and intense prostration; and the diarrhœa recurred. However, the patient's dread of chloroform and of the knife was so great that he did not consent to undergo an operation until his condition had become almost hopeless. The operation was performed at noon on March 28th, *i.e.*, the twenty-first day in hospital and forty-fifth day of his illness. From the physical signs, which were best marked in the axillary and infra-spinous part of the scapular region, it was evident that the gangrenous portion of lung was on a level with the mammary region (from the third to the sixth ribs), and that the most suitable place for incision and drainage would be between the fifth and sixth ribs in the axillary area. Accordingly, a horizontal incision two inches long was made in the fifth intercostal space of the left side, which was followed by the escape of a small quantity of fœtid purulent fluid and *débris*. By the finger it was ascertained that the axillary part of the pleural cavity corresponding to the area of gangrenous lung had been shut off by adhesions from the rest of the pleural sac. On exploring with a Wheelhouses's straight-grooved urethrotomy staff the instrument was found to pass far into the lung, so as almost to touch the pericardium; in fact, the impulse of the cardiac systole was transmitted to the staff with startling force and distinctness. Portions of gangrenous lung and pleura-*débris* and blood-clot were removed with the aid of the fingers, dressing forceps, and a long Volkmann's spoon. A drainage-tube four inches long and three-quarters of an inch in diameter was introduced, and an absorbent antiseptic dressing was applied. The drainage-tubes were gradually shortened and replaced by tubes of smaller calibre, the last of which was finally removed on April 21st, and was quite healed on the 30th. The patient's health rapidly improved, and he gained ten and a half pounds in weight between April 21st and May 8th. When he was discharged from hospital on the latter date, the following note was recorded: "The percussion note is clear and slightly hyper-resonant in the left axillary region. The breathing is harsh, and an occasional moist sound is audible near the incision scar in the fifth space. The lung is soundly healed, and he may leave hospital." The patient's mother, who had recently been operated on by Surgeon-Captain Moir, on September 3rd told him that her son resumed work exactly a month after leaving hospital, and that he was then away on a shooting expedition. Case 2: Liver and lung abscess; operations; recovery. A man aged 34 years, a slim Eurasian of poor physique, was admitted to hospital on January 27th, 1896, complaining of fever, cough, pain in the liver, and enlargement of the spleen. From January 3rd to 13th, 1896, he was treated in the General Hospital, Calcutta, for hepatitis, and was discharged apparently cured, but he returned on January 27th with the symptoms detailed above. In the beginning of February the hepatic pain disappeared, and the temperature remained normal until the fourth, when it rose to 101 deg. On the 21st he began to cough up pus, owing to a liver abscess having burst into the right lung. As many cases have been recorded in which recovery has followed the discharge of a liver abscess into either the lung or the bowel, and as there were no unfavourable symptoms, it was decided that no operation was necessary. In March, however, his symptoms became unfavourable, and gave cause for anxiety. On March 13th, after using the exploring needle, a horizontal incision two and a half inches long was made in the sixth intercostal space two and a half inches below the right nipple. On pushing the finger through a thickish layer of liver substance the abscess cavity was reached. Ten ounces of pus were evacuated, and a large-sized drainage-tube was inserted. It was not possible to palpate the aperture of communication through the diaphragm into the lung. On the next day his temperature was normal, and he had neither cough nor expectoration. During the first week of April the cough again became troublesome, and the sputa were viscid, greenish, and nummular. Later his feet became œdematous, he suffered from exhausting sweats and restless nights, and his appetite failed. The physical signs indicated excavation of the right lung in the axilla and about the mammilla. Accordingly, on May 5th, an incision was made far back in the right axilla over the fifth rib, one inch of which was resected. After puncturing the lung and dilating the passage a small cavity was found which extended upwards and inwards above the level of the nipple. This communicated with another cavity, below the nipple, which led down to the diaphragm. By introducing a metal bougie, the curve of which was convenient, a counter opening was made in front in the fourth space below the nipple and above the scar of the incision into the liver abscess. Some lung *débris*, pus, and blood were evacuated from the lower cavity in the lung. Through the axillary incision a drainage tube over three inches long and half an inch in diameter was introduced into the upper cavity. A drainage tube nearly five inches long and three-fourths of an inch in diameter was passed in from the anterior incision, through the lower cavity and towards the lateral incision. During the latter half of July he improved rapidly in every way, so much so that he was fit to be discharged from hospital and to be sent to the Eden Sanatorium at Darjeeling on the 29th. Since the patient left the hospital Surgeon-Captain Moir has had good accounts of him from time to time. The last report, dated September 9th, was from the Assistant-Surgeon in charge of the Eden Sanatorium, who stated that "he is doing very well, has no bad symptoms of any kind at pres nt, and weighs 127 lb."

Surgeon-Captain Moir, reg ·ding the statistics of operation for gangrene of the lung, says that Dr. Pendleton Dandridge makes the following statement: "Of the result in gangrenous cases Lopez reports seventeen cases of gangrene treated by opening the cavity followed by ten cures, six deaths, and one 'unknown'; Hofmokl, twenty-four cases, with seven cures, one unimproved, thirteen deaths, and two unknown, whilst in one a fistula remained."

We have quoted these cases somewhat fully that the reader may be reminded of the general clinical conditions which may be associated with the occurrence of acute pulmonary affections calling for surgical interference, conditions which are now being more widely recognised in time for active surgery to afford relief. Thus Ewart and Marmaduke Shield[16] are able to record a case of acute pulmonary gangrene in a healthy youth which resulted in pyo-pneumothorax, with severe symptoms. The case was one of those in which the gangrene is very superficial, when, as was first pointed out by Corbin (Journ. Hebd. de Méd., 1830, VII., p. 126), consecutive pleurisy, if accompanied by pyo-pneumothorax, is very common. Chloroform having been administered, an incision was made into the left pleural cavity through the sixth intercostal space immediately behind the mid-axillary line. A large amount of very fœtid pus and gas escaped and an empyema tube was inserted. At the end of the operation it was found that the heart's apex beat had returned to its normal position. On the 29th, after a fair night, with very little cough and little expectoration, the patient's breathing was still rapid, but his general condition had much improved. On dressing the wound a large quantity of very fœtid pus came out. The amount of discharge steadily diminished, the fœtor became less marked, and he rapidly improved in health. Dr. Ewart remarks that the points of interest in this case were the patient's early age, healthy antecedents and surroundings, and the absence of any clue as to the source of infection or of septic influence; the changes in the physical signs, which led to the case being viewed in succession as one of broncho-pneumonia, of empyema, of bronchiectasis, and of gangrene with pneumothorax; the almost desperate condition of the patient at the time of the operation; and, finally, the complete recovery, thanks to the latter.

[15] Lancet, Jan. 9, 1897. [16] Lancet, June 19, 1897.

